# Local Antibiotics in the Treatment of Diabetic Foot Infections: A Narrative Review

**DOI:** 10.3390/antibiotics12010124

**Published:** 2023-01-09

**Authors:** Laura Soldevila-Boixader, Alberto Pérez Fernández, Javier Muñoz Laguna, Ilker Uçkay

**Affiliations:** 1 Infectiology, Unit for Applied and Clinical Research, Balgrist University Hospital, 8008 Zurich, Switzerland; 2 Infectious Diseases Department, Bellvitge University Hospital, 08907 Hospitalet de Llobregat, Barcelona, Spain; 3 Orthopedics Department, Head of the Foot and Ankle Unit and Coordinator of Diabetic Foot Unit, Bellvitge University Hospital, 08907 Hospitalet de Llobregat, Barcelona, Spain; 4 EBPI-UZWH Musculoskeletal Epidemiology Research Group, Balgrist University Hospital, University of Zurich, 8008 Zurich, Switzerland; 5 University Spine Centre Zurich (UWZH), Balgrist University Hospital, University of Zurich, 8008 Zurich, Switzerland

**Keywords:** diabetic foot infection, osteomyelitis, local antibiotics, topical antibiotics, local delivery antibiotic systems

## Abstract

Along with the increasing global burden of diabetes, diabetic foot infections (DFI) and diabetic foot osteomyelitis (DFO) remain major challenges for patients and society. Despite progress in the development of prominent international guidelines, the optimal medical treatment for DFI and DFO remains unclear as to whether local antibiotics, that is, topical agents and local delivery systems, should be used alone or concomitant to conventional systemic antibiotics. To better inform clinicians in this evolving field, we performed a narrative review and summarized key relevant observational studies and clinical trials of non-prophylactic local antibiotics for the treatment of DFI and DFO, both alone and in combination with systemic antibiotics. We searched PubMed for studies published between January 2000 and October 2022, identified 388 potentially eligible records, and included 19 studies. Our findings highlight that evidence for adding local antibiotic delivery systems to standard DFO treatment remains limited. Furthermore, we found that so far, local antibiotic interventions have mainly targeted forefoot DFO, although there is marked variation in the design of the included studies. Suggestive evidence emerging from observational studies underscores that the addition of local agents to conventional systemic antibiotics might help to shorten the clinical healing time and overall recovery rates in infected diabetic foot ulcers, although the effectiveness of local antibiotics as a standalone approach remains overlooked. In conclusion, despite the heterogeneous body of evidence, the possibility that the addition of local antibiotics to conventional systemic treatment may improve outcomes in DFI and DFO cannot be ruled out. Antibiotic stewardship principles call for further research to elucidate the potential benefits of local antibiotics alone and in combination with conventional systemic antibiotics for the treatment of DFI and DFO.

## 1. Introduction

Diabetic foot infections (DFI), including diabetic foot osteomyelitis (DFO), represent a societal challenge associated with substantial morbidity, prolonged hospitalizations, prospective amputations, and higher overall healthcare costs [1]. Diabetes prevalence varies between countries of different economic levels, being generally higher in low-income countries (12.3%) than in high-income countries (6.6%), although these differences are not easily explained by the distribution of conventional risk factors [2]. In general, systemic antibiotics are recommended as a reference treatment for infected diabetic feet [3,4], while local antimicrobial agents and surgically implanted delivery systems have been overlooked. Thus far, local antibiotic therapies have been studied within the context of non-diabetic orthopedic infections and orthopedic trauma, mainly as antibiotic-containing beads and antibiotic-containing cement. These interventions have targeted the treatment of osteomyelitis, and the prevention and therapy of implant infections [5,6]. In the management of an infected diabetic foot, clinicians often encounter three scenarios in which local antibiotic use could be advantageous: (1) infected diabetic foot ulcers (DFU), (2) soft tissue infections without DFO, and (3) DFO. The emergence and attractiveness of local antibiotics for DFI and DFO rely on the opportunity they offer to reduce the extent of antimicrobials needed, their ability to achieve high antibiotic concentrations in the localized infected area, their limited systemic absorption and reduced adverse events, and their evident safety in relation to multidrug resistance [7,8]. The disadvantages are few and may include local skin hypersensitivity or contact dermatitis, prolonged wound discharge, the relative burden associated with frequent reapplications over time, and the possible unintended contamination of opened multidose containers [8].

It is worth mentioning that in the local treatment of DFI and DFO, aminoglycosides and vancomycin are the most studied antibiotics, having both broad-spectrum activity and being able to be incorporated into any delivery vehicle. The biological properties of these two antibiotics also allow them to remain thermostable during the exothermic reaction that occurs during the polymerization of the cement in local applications, ultimately delivering a significantly higher concentration locally than via the blood system [9]. 

### 1.1. Topical Antibiotics in Diabetic Foot Ulcers and Postoperative Wounds in a Diabetic Foot

**Figure 1:**
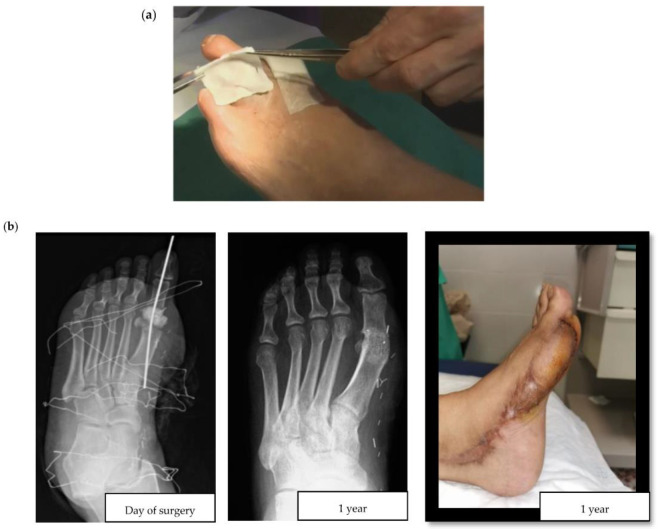
(**a**) Application of a collagen sponge on a diabetic foot wound (courtesy of Dr. Ilker Uçkay)**;** (**b**) Clinical case of a hallux valgus osteomyelitis with bone resection, filling with Cerament G^®^ and a flap. We can observe bone osteointegration 1 year later (courtesy of Dr. Alberto Pérez Fernández).

For the purposes of this review, we consistently apply the term topical to refer to the local administration of antibiotics through an infected diabetic foot ulcer (DFU). Topical antibiotic interventions have previously been studied in the assessment of the bioburden and biofilm of diabetic foot wounds [10]. For example, an in vitro experiment compared the efficacy of topical vancomycin and gentamicin to systemic antibiotics for the eradication of polymicrobial biofilms. The authors observed a bioburden reduction of 5 and 8 logarithms (colony forming units per milliliter), a finding with uncertain yet promising clinical relevance, and supportive of further studies to reveal the usefulness of topical antibiotic strategies in the surgical treatment of DFIs [11]. When considering the multiple topical antimicrobials available, it is also worth highlighting that a recent high-quality systematic review failed to show the superiority of any particular topical antimicrobial [12]. This lack of superiority could be partially explained by the local agent choices, encompassing a wide range of antiseptics, antibiotics, and antimicrobial dressings. More consistency in design is seen in clinical studies of local agents in DFIs since the gentamicin collagen sponge and the superficial pexiganan peptide (Figure 1a) tend to be incorporated. 

Interestingly, in vitro studies have shown that the kinetics of antibiotic release from gentamicin collagen sponge matrices can reach 95% in as little as 1.5 h [13,14]. A plausible explanation is that collagen may produce scaffolds for fibrin deposition, resulting in the healing of tissue defects and the acceleration of wound healing. It is noteworthy that the success of gentamicin collagen sponges has been documented since 1997 [15]. For its part, pexiganan, an antimicrobial synthetic peptide and analogue of the magainin peptide, has shown similarly promising in vitro results, as judged by the susceptibility of bacteria isolated from the infected diabetic foot to pexiganan in 1999. However, the first therapeutic experience with gentamicin in an infected diabetic foot did not occur until 2008 [16,17]. 

### 1.2. Local Delivery Antibiotic Systems in Operated Diabetic Foot Osteomyelitis 

There are two major challenges associated with DFO surgeries: postoperative wound healing in the operated, and ischemia triggered by the residual death space of the former bone. In DFO treatment, two main types of antibiotic delivery system materials are currently available: non-absorbable and absorbable. According to the explored literature, the most frequently used non-absorbable material in DFO is polymethylmethacrylate (PMMA)—an acrylic used extensively in orthopedic surgery for chronic osteomyelitis and implant-related infections [18,19]. Some of the most common antibiotic-loaded commercial PMMA cements include Cemex^®^, Simplex^®^, Eurofix^®^, Palacos^®^, Copal^®^, and Refobacin^®^. As with all non-absorbable materials, the main disadvantage of PMMA is its surgical removal, resulting in additional intervention following the release of all drugs. Alternatively, there are hybrids of biodegradable carrier systems that take advantage of different properties to improve local antibiotic release. They have different presentations, such as Cerament G/V^®^ (see Figure 1b for a clinical case example) or Stimulan^®^ [20]. The development and use of contemporary absorbable biomaterials is an area of ongoing advancement. These materials have some advantages compared to PMMA, including better osteointegration and the lack of need for surgical removal. In this respect, the importance of considering the composition of these materials should also be highlighted, especially as to whether or not they contain hydroxyapatite, which is highly osteoconductive and promotes bone ingrowth. For example, the unique ratio of hydroxyapatite and calcium sulphate in Cerament^®^ makes it particularly suitable for absorption and stimulation of new bone formation at the same rate. In contrast, Stimulan^®^ only contains hemihydrate calcium sulfate, which retains its absorbable properties, but sacrifices osteoconductivity and bone growth. 

Table 1 summarizes the key properties of the three classes of commercial antibiotics, based on their local delivery systems. Other delivery systems are mainly used for long bones, and only exceptionally in the infected diabetic foot.

In this narrative review, we summarized key relevant observational studies and clinical trials on local antibiotics (topical and intraosseous) for the treatment of bacterial DFI and DFO, both alone and concomitant to systemic antibiotics. Because some systematic reviews have been published without any definitive conclusions regarding the use of local antibiotics in DFI and DFO [21], we believe that an expert-informed pragmatic narrative review could help identify a potential research gap in this field, informing future research efforts. Furthermore, our choice of study design is also motivated by the desire to engage clinicians in the evolving fields of DFI and DFO. Importantly, in this narrative synthesis, we refrained from exploring the prophylactic properties of antimicrobial agents in DFI and DFO for pathogens other than bacteria, or outside the diabetic foot, for which the reader might consider other articles. 

## 2. Results

We identified 388 potentially eligible studies following our search strategy. All studies were screened and assessed for eligibility by the lead author (LSB), and only those that met the eligibility criteria were included in the analysis. 

19 studies were finally included in our review: 5 randomized clinical trials (RCTs) related to topical antibiotics, and 14 studies (1 RCT and 13 retrospective studies) related to local delivery antibiotics in DFI and DFO. In general, retrospective studies exploring local antibiotic delivery had methodological shortcomings and included highly heterogeneous study populations. Hence, most studies lacked comparability with each other and were not suitable for formal quantitative pooling via meta-analysis (Table 2). A remarkable finding emerging from our included RCTs is that none of them used topical agents on infected and intact skin (i.e., erysipelas of the diabetic foot). 

### 2.1. Topical Antibiotics in Infected Diabetic Foot Ulcers

Our pragmatic narrative synthesis stressed that chronic open wounds of the diabetic foot are often complicated by infection and, together with advanced ischemia, can result in amputations [22]. Some of the included studies underlined that topical treatment has the advantage of avoiding systemic adverse effects, providing a higher concentration of the target site, and allowing the use of different antibiotics, a finding with potential relevance informing future DFI-related antibiotic stewardship programs [8]. 

#### 2.1.1. Postsurgical Wound

Varga et al. [23] performed an RCT to explore the efficacy of the application of gentamicin collagen sponges to postsurgical wounds in diabetic patients undergoing minor amputation. The median duration of wound healing duration in the gentamicin sponge group was 3.0 weeks (range: 1.7 to 17.1 weeks) compared to 4.9 weeks (range: 2.6 to 20.0 weeks) in the control group, a between-group difference that was deemed statistically significant (*p* < 0.05). 

#### 2.1.2. Topical Antibiotics in Mild DFU Infections

One of the included multicenter RCTs, consisting of two consecutive double-blind trials, compared topical versus systemic antimicrobial therapy for the treatment of mildly infected DFU [17]. A total of 835 patients were randomized to either pexiganan cream or oral ofloxacin for mild DFU infection. No between-group differences were found regarding the proportion of patients achieving clinical improvement (85% vs. 90%), or microbiological eradication (42% vs. 47%). Moreover, the incidence of worsening cellulitis and amputations was similar between the two arms (2% vs. 4%) [17]. 

Another relevant pilot RCT for mild DFI with ulcers (n = 22), performed within the Swiss context, compared local gentamicin sponges to standard wound care without systemic antibiotics. At the primary endpoint, 20 patients (91%) were considered to have reached a clinical cure for infection, and 2 (9%) were deemed to have achieved significant improvement. In terms of microbiological outcomes, only 12 patients (56%) achieved microbiological eradication of all pathogens. This study did not find any significant between-group differences in either clinical or microbiological outcomes, yet it provided reassurance about the tolerability and feasibility of adding gentamicin-sponges to standard wound care [24].

#### 2.1.3. Topical Antibiotics in Moderate-Severe DFU Infections 

A pragmatic RCT for moderate to severe DFI (n = 56) compared a topical gentamicin collagen sponge plus a systemic antibiotic intervention versus systemic antibiotics alone. No significant between-group differences were observed in the main outcomes. However, this study is not directly comparable to the other included studies in our narrative review due to the design of the combined local and systemic antibiotic intervention and the underlying research question of interest [25]. 

A similar RCT based in Switzerland compared gentamicin-collagen sponges in combination with systemic therapy to standard wound care with systemic therapy in 88 diabetic patients with moderate to severe DFIs. Although the gentamicin sponge intervention was well tolerated, it did not show superiority to standard wound care. Nonetheless, a suggestive trend toward more rapid and pronounced ulcer healing was observed in the gentamicin-sponge during the second half of the study (from 3 to 5 weeks) [26].

### 2.2. Local Delivery Antibiotic Systems for Diabetic Foot Osteomyelitis 

#### 2.2.1. Local Delivery Systems for Forefoot Osteomyelitis

The reviewed body of evidence regarding local delivery systems for forefoot DFO is limited to one RCT. Forefoot DFOs have an inherent higher frequency of therapeutic surgical amputations and are always at risk of postoperative skin breakdown and subsequent major amputations [27,28]. In clinical care and based on the expert clinical opinion of the authors, surgeons tend to ignore whether all infected bone has been completely resected and rely on long-term postoperative antibiotic interventions. Armstrong et al. [29] explored the potential usefulness of antibiotic-impregnated calcium sulfate beads for the local treatment of deeply infected DFUs, both in the presence and absence of osteomyelitis and surgery. The authors suggest that local delivery systems may be a promising addition to the therapeutic arsenal in the future. However, up to this point, there is no high-quality evidence to support their use. Furthermore, Krause et al. [30] published a retrospective study of 60 forefoot DFO patients exposed to systemic antibiotics and tobramycin beads versus without and found no differences between groups in primary outcomes. 

Niazi et al. [31] published the largest multicenter study on local adjuvant antibiotics in patients with DFU, enrolling 70 participants. The intervention of interest involved cerement G, an antibiotic-loaded absorbable calcium sulfate/hydroxyapatite biocomposite which showed promising results when added to conventional systemic treatment. For its part, Chatzipapas et al. [32] compared surgical treatment, with and without local antibiotic administration, for DFO in a three-arm RCT of 25 participants, revealing that adjunctive local antibiotic therapy failed to improve the overall remission rates. Other similar small studies have been published on the use of local antibiotic beads and antibiotic spacers, showing null results [33,34,35,36,37,38,39,40]. These studies have systematically failed to show superiority in cure proportions when used concomitantly with systemic antibiotic agents, or simply could not claim superiority due to the lack of an appropriate comparator group.

In contrast, one small RCT of 36 participants with diabetic foot ulcers showed that coadjuvant local antibiotics helped shorten healing times [41]. Similar findings were observed in a retrospective study involving 32 DFU patients [42].

#### 2.2.2. Local Delivery Antibiotics for Midfoot and Hindfoot Osteomyelitis

Conservative treatment in midfoot-hindfoot DFO is less frequently investigated than forefoot DFO [43]. That said, when compared to forefoot DFO, midfoot-hindfoot DFO tend to have a less favorable prognosis, given that the probability of undergoing major amputation has been shown to be considerably higher (midfoot-hindfoot: 20% to 46% vs. forefoot: 6%) [44]. Pertinent to this part of the narrative synthesis, Drampalos et al. [45] described a series of 12 patients with calcaneal DFO who underwent calcaneal resection and local delivery of gentamicin antibiotics. At 16 weeks of infection, calcaneal DFO was eradicated in all 12 patients with a single-stage procedure following a bone-preserving technique. Nevertheless, this study, based in the United Kingdom, had no control group, which seriously limited the inferences that could be derived from it.

**Table 2:** Study characteristics of trials and observational studies examining topical and local delivery antibiotics for diabetic foot infection or diabetic foot osteomyelitis.

Topical Antibiotics					
First Author, Year Country	Study Design	Interventions	Study Population (Sample Size, n); Microbiology *	Narrative Summary of Main Findings	Outcome Quantitative Estimates
**Lipsky, 2008 [17]** **USA**	RCT	A: Pexiganan cream vs. B: ofloxacin 400 mg daily	Participants with mildly infected diabetic foot ulcers (n = 835); study 303 (n = 493); study 304 (n = 342) *Staphylococcus aureus*, *Enterococcus faecalis*, *Streptococcus agalactiae*	Pexiganan cream and ofloxacin showed similar clinical improvement proportions as ofloxacin	**Clinical cure or improvement** A vs. B, study 303 (85% vs. 91%); between-group difference: −6.04 (−11.74 to −0.33) A vs. B, study 304 (89.5% vs. 89.5%); between-group difference: 0.00 (−6.51 to 6.51)
**Lipsky, 2012 [25]** **USA**	RCT	A: Gentamicin-collagen sponge vs. B: standard treatment **	Participants with mildly diabetic foot ulcers infected (n = 56) CNS, MS *S. aureus*, and MR *S. aureus*	Gentamicin-collagen sponge group showed higher proportion of clinical cure	**Clinical cure** A vs. B, 22/22 (100%) vs. 7/10 (70%) ^¶^
**Varga, 2014 [23]** **Czech Republic**	RCT	A: Gentamicin-collagen sponge vs. B: standard treatment ^†^	Diabetic patients following minor amputation (n = 50) *S. aureus*, *E. faecalis*, *Klebsiella* spp.	There was a tendency to faster healing in the gentamycin-sponge group	**Time to wound healing** A vs. B, 3 weeks (1.7 to 17.1 weeks) vs. 4.9 weeks (2.6 to 20.0 weeks) ^¶^
**Uçkay, 2018 [26]** **Switzerland**	RCT	A: Gentamicin-collagen sponge plus systemic antibiotics vs. B: systemic antibiotics	Patients with moderate-to-severe diabetic foot ulcers infected (n = 88) *S. aureus*, *Streptococcus* spp., *Escherichia coli*	Gentamycin-sponge was well tolerated and trended toward shorter healing time, but did not reach statistical significance in clinical cure	**Clinical cure** A vs. B, 31/43 (72%) vs. 26/45 (58%), *p* = 0.16
**Uçkay, 2018 [24]** **Switzerland**	RCT	A: Gentamicin-collagen sponge vs. B: standard treatment	Patients with mild diabetic foot infection (n = 22) *S. aureus*, *P. aeruginosa*, *Staphylococcus epidermidis*	Gentamycin-sponge was well tolerated, but did not reach statistical significance in clinical cure	**Clinical cure** A vs. B, 10/11 (91%) vs. 10/11 (91%), *p* = 1.00.
**Local delivery antibiotics**					
**First Author, Year** **Country**	**Study Design**	**Interventions**	**Study Population (Sample Size, n)**	**Narrative Summary of Main Findings**	**Outcome** **Quantitative Estimates**
**Krause, 2009 [30]** **Canada**	RS	TMA in diabetic foot using A: antibiotic beads (tobramycin-impregnated CaSO_4_ beads) vs. B: standard treatment	Patients with forefoot amputation (n = 60) MR *S. aureus*, MS *S. aureus*, *Pseudomonas aeruginosa,* and *E. coli*	Antibiotic beads may be a useful addition to TMA for patients with non-healing diabetic ulcerations of the forefoot, although no differences were observed in the final transtibial amputation	**Transtibial amputation** A vs. B, 13/49 (27%) vs. 4/16 (25%)
**Roukis, 2010 [40]** **USA**	CS	Debridement of soft tissue and bone plus PMMA antibiotic. Days later, another surgery was performed with a bone graft and fasciocutaneous flap.	DFIs (n = 15) MR *S. aureus* (in 2 patients)	Overall, clinical cure proportion was satisfactory and TMA remained low	**Clinical cure and amputation** 9/15 (60%) were cured and 2/15 (13%)
**Melamed, 2012 [36]** **Israel**	CS	PMMA and gentamicin/ vancomycin-impregnated	Patients with severe forefoot DFO (n = 23) NR	Most patients achieved clinical cure	**Clinical cure, time to clinical cure and amputation** 21/23 (91.3%), 21.1 months, and 2/23 (8.7%)
**Dalla Paolla, 2015 [33]Italy**	RS	After surgical debridement with removal of the infected bone, vancomycin-gentamicin-impregnated bone cement was inserted into the surgical site.	Diabetic patients with 1st ray osteomyelitis (n = 28) NR	Low relapse of ulcer recurrence	**Ulcer recurrence** 4/28 (14%)
**Jogia, 2015 [35]** **UK**	CS	CaSO_4_, vancomycin, and gentamicin locally	Patients with DFO (n = 20) NR	All patients achieved a satisfactory time to clinical cure, and no recurrence was observed	**Time to clinical cure** The median was 5 weeks.
**Elmarsafi, 2017 [34] USA**	RS	PMMA with gentamycin, vancomycin or tobramycin	Patients with DFO (n = 27) NR	More than a fourth of the participants required amputation of the ipsilateral foot, although they were considered not directly related to the use or removal of the spacer	**Amputation** 26.7% partial foot amputation of the ipsilateral foot
**Drampalos, 2018 [45] UK**	RS	CaSO_4_, hydroxyapatite, and gentamycin locally	Chronic calcaneal osteomyelitis (n = 12) *S. aureus*, *E. coli*, *P. aeruginosa*	The wound healed in all patients in a single-stage procedure.	**Time to clinical cure** Mean (range) 16 weeks (12 to 18 weeks)
**Qin, 2019 [37] China**	RS	A: Vancomycin and/or gentamycin impregnated CaSO_4_ beads after bone resection vs. B: bone resection alone.	Patients with DFO (n = 46, 18 vs. 28) MS *S. aureus*, *E. coli*, *E. faecalis*	Local antibiotics prevented the recurrence of DFO but did not improve the time to clinical cure.	**Time to Clinical cure** A vs. B, 13.3 vs. 11.2 weeks **Amputation** A vs. B, 7% vs. 0%.
**Niazi, 2019 [31] UK**	CS	Gentamycin-impregnated CaSO_4_/hydroxyapatite bio-composite along with surgical debridement and systemic antibiotics.	Patients with DFO (n = 70) MS *S. aureus*, MR *S. aureus*, *Streptococcus* spp.	High proportion of infection eradication, and acceptable ulcer healing time	**Clinical cure** 90% of patients Ulcer healing time (mean): 12 weeks.
**Mendame Ehya RE, 2021 [41] China**	RCT	A: Antibiotic-loaded bone cement (with vancomycin, cefoperazone or gentamicin) vs. B: vacuum-assisted closure	Patients with DFU (n = 36, 18 in every arm) *S. aureus*, *P. aeruginosa*, *E. coli*	The wound healing time was shorter for the antibiotic-loaded bone cement group.	**Time to Clinical cure** A vs. B, 79 vs. 102 days
**Patil P, 2021 [39] India**	RS	CaSO_4_ beads with several antibiotics	Patients with DFI (n = 106) *P. aeruginosa*, *Klebsiella pneumoniae*, *E. coli*	The use of locally released antibiotics from synthetic calcium sulfate may offer benefits in the DFU infection, although there was marked variability in time to clinical cure	**Time to Clinical cure** 47–90 days
**Chatzipapas, 2022 [32] Greece**	CS	A: surgical debridement plus systemic antibiotic vs. B: surgical debridement plus local PMMA vs. C: surgical debridement plus antibiotic-loaded hydroxyapatite and CaSO_4_ sulfate beads.	Patients with forefoot and calcaneal DFO (n = 25) *Staphylococci* spp. (in 11 patients)	All healing parameters improved in both local antibiotic groups, but there were no between-groups differences in clinical cure proportions overall.	**Clinical cure** A: 87.5%, B: 100%, and C: 87.5% at 17, 18, and 19 weeks, respectively.
**Sun YW, 2022 [42] China**	RS	A: Gentamicin, vancomycin or cefoperazone-sulbactam (local antibiotics) with /without systemic antibiotics vs. B: vaccum-assisted closure	Patients with DFU (n = 32) *S. aureus*, CNS, *Streptococcus* spp.	Patients in the study group recorded a shorter healing time compared with the control group	**Time to Clinical cure** A vs. B, 44 ± 17 vs. 64 ± 30 days
**Morley R, 2022 [38] UK**	RS	CaSO_4_ with gentamicin and vancomycin with/without systemic antibiotics	Mostly patients with DFI (n = 137, with 113 DFI) NR	Although clinical cure proportion was satisfactory, healing time was significantly increased for the comorbidities of diabetes and vasculopathy, and for those requiring prolonged systemic postoperative antibiotics	**Clinical cure** 82.5%, with a healing time (mean): 11.3 weeks

Abbreviations: CaSO_4_, calcium sulfate; CNS, coagulase-negative Staphylococci; CS, case series; DFI, diabetic foot infections; DFO, diabetic foot osteomyelitis; DFU, diabetic foot ulcer; NR, microbiology not reported; MR, methicillin-resistant; MS, methicillin-sensitive; PMMA, polymethylmethacrylate antibiotic-loaded bone cement spacer; RCT, randomized controlled trial; RS, retrospective study; TMA, trans metatarsal amputation. * Main bacteria inducing infection in order of frequency. ** All patients received systemic antibiotics. ^¶^ Statistically significant, *p* < 0.05. ^†^ Some patients received systemic antibiotics.

## 3. Discussion

### 3.1. Statement of Major Findings

Our narrative review underscores that there is very limited evidence favoring the addition of local antibiotic delivery systems to standard DFI and DFO treatments. Experimental and observational studies of local antibiotic interventions published to this date have more frequently targeted forefoot DFO, although the marked heterogeneity of the study populations in the included studies precludes the drawing of robust general statements. Importantly, some of the included studies in our narrative synthesis reveal that the addition of local agents to conventional systemic antibiotics might help in key outcomes of a diabetic foot, namely time to recovery and clinical cure proportion. Critically, interventions consisting of local antibiotics as a standalone approach remain neglected in DFI and DFO management.

### 3.2. Meaning and Importance of Findings

Our review identifies some key knowledge gaps in the rapidly evolving area of DFI and DFO treatment and supports further research on local antibiotics. It is worth emphasizing that, given that some of the included clinical trials were likely underpowered, the lack of statistically significant results should not be interpreted as strong evidence against the clinical usefulness of local antibiotics. Furthermore, because larger-scale and appropriately powered trials have focused on comparing the addition of topical or local intraosseous antibiotics to conventional systemic antibiotics—which tend to include professional wound care and adequate off-loading [17]—alternative research questions and causal estimands are worth exploring in the near future. In addition, multiple observational studies in our narrative synthesis lacked a comparator group, severely compromising the validity of their estimates. Nonetheless, diabetic foot patients exposed to local antibiotics show a satisfactory evolution in many of the studies in this review, and the possibility that they are less likely to undergo major amputations and less prone to experience other severe outcomes cannot be ruled out. Hence, identifying the subgroup of diabetic foot patients more likely to benefit from local antibiotics will remain a research priority for clinician scientists in the area of DFI and DFO, and an area of relevance for population health considering antibiotic stewardship principles. 

### 3.3. Findings in the Context of Existing Reviews and International Guidelines

Only recently has antibiotic management found its way into the field of academic research, motivated primarily by questions related to the ideal duration of systemic antibiotic therapy [46,47,48]. However, it is worth mentioning that other relevant reviews on local antibiotics and wound healing following surgery for foot infections in diabetics, concluding that local antibiotics should not be used due to the lack of evidence [21]. Another relevant high-quality Cochrane systematic review on local agents in the diabetic foot showed no differences between the various topical agents, including antibiotics and antiseptic agents [12]. Given the seemingly attractive risk–benefit ratio of local delivery systems in the management of DFOs, our findings align more closely with those of a review published in 2015, which recommended and made a call to action for future RCTs to elucidate a new approach on how local delivery systems can be integrated into the overall therapeutic strategy for operated DFOs [49].

Remarkably, the international Infectious Diseases Society of America (IDSA) and International Working Group of Diabetic Foot (IWGDF) guidelines have a strong position against the use of topical antimicrobials in the diabetic foot, although they also promote research in this promising field. IDSA justifies its position based on limited data on topical antimicrobials for mild diabetic foot infections with minimal cellulitis [3,17], while IWGDF also points to the lack of convincing data for standard use. Future research may influence the dynamic nature of these reference guidelines, ultimately creating alternative strength of recommendation classifications to guide clinical decision making in particular DFI and DFO subgroups. 

### 3.4. Relevance and Implications for Future Research

DFIs are associated with significant therapeutic challenges, and decisions regarding their optimal therapeutic approach are difficult. In addition to surgical debridement, professional wound care, adequate off-loading, and eventual revascularization, systemic targeted antibiotics have traditionally been required when there is an infection, especially for moderate and severe DFIs.

With clinical equipoise being justifiable, future trials should strongly consider head-to-head comparisons of local antimicrobial therapy alone versus systemic therapy in certain subsets of DFI and DFO (i.e., localized DFIs in the forefoot and infected DFU). It is plausible that, in addition to shortening the treatment of DFO, topical antibiotics could be effective in mild and ulcerated DFI. A specific and potentially informative trial in the future should contemplate assessing short-term (1 to 2 weeks) oral therapy versus topical antibiotic interventions in a study population of mild diabetic foot ulcer infections or diabetic foot ulcers with secondary signs of infection. Although clinical studies of local antibiotics for DFO are more difficult to justify, some valuable information could be revealed by targeting specific subpopulations and carefully refining research questions of interest. For example, local antibiotics trials for DFO would benefit from homogeneous eligibility criteria, well-defined anatomical locations, specific surgical approaches, and consistent primary endpoints.

### 3.5. Limitations

The main limitations of this study arise from the narrative review design and the pragmatic search strategy, which may introduce subjectivity in the selection of studies. However, we judiciously and purposively selected clinical and observational evidence to reflect the relevant body of evidence to answer our research question and provided an expert interpretative overview of the field. 

Within the context of our review and having made suggestions for further research, it is worth citing a promising published protocol for a multicenter trial of orthopedic infection, which will use a non-inferiority design to compare local antibiotics plus 7 or less days of systemic therapy versus local antibiotics plus 4 or more weeks of systemic therapy. Remarkably, this upcoming trial will include adults undergoing surgical treatment for orthopedic infections, including a diabetic foot, and the surgical intervention will consist of a local antibiotic-carrier implantation at the site of infection [50]. Novel protocol designs for future studies highlight the importance of coordinated efforts to help surgeons and physicians be able to gain collective and personal knowledge on optimal DFI and DFO management approaches, all while improving patient-centered care and promoting evidence-based principles.

## 4. Materials and Methods

We performed a literature review in the PubMed database in October 2022, searching for the following terms: (diabetic foot) AND (local antibiotics OR topical antibiotics OR calcium sulfate impregnated OR antibiotic delivery OR bone cement). We limited our pragmatic search strategy to one database for feasibility purposes, as we considered that this would allow us to answer the research question of interest given our resource constraints. We applied filters to restrict potentially eligible titles to be published between January 2000 and October 2022.

To be included, studies needed to enroll adult patients ≥ 18 years with ongoing diabetic foot problems (diabetic foot ulcers, soft tissue infections, or diabetic foot osteomyelitis) and local antibiotic use. We included randomized clinical trials, observational studies, and case series with > 10 patients. In addition, we hand-searched for additional citations from the references of the retrieved articles. We excluded articles written in languages other than English. Further details on our search strategy can be found in the Supplementary Materials (Figure S1 and File S1). 

In preparing this narrative review, we adhered to key applicable items for narrative reviews from the PRISMA statement [51]. We also followed the recommendations of a proposed scale for the quality assessment of narrative reviews [52].

## 5. Conclusions

Topical and local intraosseous antibiotics might improve the healing of mild DFU infections or operated DFO cases, although they have been explicitly neglected as standalone interventions. Most studies published to date have failed to show the superiority of adding these local treatments to systemic antibiotic administration. Given the potential systemic antibiotic-sparing benefits of local antibiotics as standalone treatments, further research is needed to promote antibiotic stewardship principles and advance the field of DFI and DFO management.

## Figures and Tables

**Table 1:** Local delivery antibiotics in diabetic foot osteomyelitis.

Commercial Antibiotic Mode of Administration	Composition (%)	Absorbable	Generic Name
Cemex^®^, Simplex^®^, Eurofix^®^, Palacos^®^, Copal^®^, Refobacin^®^ **Antibiotic-loaded PMMA cement**	Polymethylmethacrylate (100%)	No	GEN/VAN/TOB or CLIN
Cerament^®^ **Injectable synthetic bone void filler**	Calcium sulphate (60%) Hydroxyapatite (40%)	Yes	GEN/VAN
Stimulan^®^ **Injectable synthetic bone void filler or beads**	Calcium sulphate (100%)	Yes	GEN/VAN/TOB

Abbreviations: CLIN, clindamycin; GEN, gentamicin; PMMA, polymethylmethacrylate; TOB, tobramycin; VAN, vancomycin.

## Data Availability

Not applicable.

## Supplementary Materials

The following supporting information can be downloaded at: https://www.mdpi.com/article/10.3390/antibiotics12010124/s1, Figure S1: Flowchart of included studies. File S1: Search strategy in PubMed.

## References

[1] Lavery L.A., Armstrong D.G., Wunderlich R.P., Mohler M.J., Wendel C.S., Lipsky B.A. (2006). Risk Factors for Foot Infections in Individuals With Diabetes. Diabetes Care.

[2] Dagenais G.R., Gerstein H.C., Zhang X., McQueen M., Lear S., Lopez-Jaramillo P., Mohan V., Mony P., Gupta R., Kutty V.R. (2016). Variations in Diabetes Prevalence in Low-, Middle-, and High-Income Countries: Results From the Prospective Urban and Rural Epidemiological Study. Diabetes Care.

[3] Lipsky B.A., Berendt A.R., Cornia P.B., Pile J.C., Peters E.J.G., Armstrong D.G., Deery H.G., Embil J.M., Joseph W.S., Karchmer A.W. (2012). 2012 Infectious Diseases Society of America Clinical Practice Guideline for the Diagnosis and Treatment of Diabetic Foot Infections. Clin. Infect. Dis..

[4] Lipsky B.A., Senneville É., Abbas Z.G., Aragón-Sánchez J., Diggle M., Embil J.M., Kono S., Lavery L.A., Malone M., Asten S.A. (2020). Guidelines on the Diagnosis and Treatment of Foot Infection in Persons with Diabetes (IWGDF 2019 Update). Diabetes Metab. Res. Rev..

[5] Haddad F.S., Masri B.A., Campbell D., McGraw R.W., Beauchamp C.P., Duncan C.P. (2000). The PROSTALAC Functional Spacer in Two-Stage Revision for Infected Knee Replacements. J. Bone. Jt. Surg..

[6] Hofmann A.A., Goldberg T.D., Tanner A.M., Cook T.M. (2005). Ten-Year Experience Using an Articulating Antibiotic Cement Hip Spacer for the Treatment of Chronically Infected Total Hip. J. Arthroplast..

[7] Markakis K., Faris A.R., Sharaf H., Faris B., Rees S., Bowling F.L. (2018). Local Antibiotic Delivery Systems: Current and Future Applications for Diabetic Foot Infections. Int. J. Low Extrem Wounds.

[8] Lipsky B.A., Hoey C. (2009). Topical Antimicrobial Therapy for Treating Chronic Wounds. Clin. Infect. Dis..

[9] Wininger D.A., Fass R.J. (1996). Antibiotic-Impregnated Cement and Beads for Orthopedic Infections. Antimicrob. Agents Chemother..

[10] Lavigne J.-P., Sotto A., Dunyach-Remy C., Lipsky B.A. (2015). New Molecular Techniques to Study the Skin Microbiota of Diabetic Foot Ulcers. Adv. Wound Care (New Rochelle).

[11] Crowther G.S., Callaghan N., Bayliss M., Noel A., Morley R., Price B. (2021). Efficacy of Topical Vancomycin- and Gentamicin-Loaded Calcium Sulfate Beads or Systemic Antibiotics in Eradicating Polymicrobial Biofilms Isolated from Diabetic Foot Infections within an In Vitro Wound Model. Antimicrob. Agents Chemother..

[12] Dumville J.C., Lipsky B.A., Hoey C., Cruciani M., Fiscon M., Xia J. (2017). Topical Antimicrobial Agents for Treating Foot Ulcers in People with Diabetes. Cochrane Database Syst. Rev..

[13] Swieringa A.J., Goosen J.H.M., Jansman F.G.A., Tulp N.J.A. (2008). In Vivo Pharmacokinetics of a Gentamicin-Loaded Collagen Sponge in Acute Periprosthetic Infection: Serum Values in 19 Patients. Acta Orthop..

[14] Kilian O., Hossain H., Flesch I., Sommer U., Nolting H., Chakraborty T., Schnettler R. (2008). Elution Kinetics, Antimicrobial Efficacy, and Degradation and Microvasculature of a New Gentamicin-Loaded Collagen Fleece. J. Biomed. Mater. Res. B Appl. Biomater..

[15] Faludi S., Kádár E., Kószegi G., Jakab F. (1997). Experience Acquired by Applying Gentamicin-Sponge. Acta. Chir. Hung..

[16] Ge Y., MacDonald D., Henry M.M., Hait H.I., Nelson K.A., Lipsky B.A., Zasloff M.A., Holroyd K.J. (1999). In Vitro Susceptibility to Pexiganan of Bacteria Isolated from Infected Diabetic Foot Ulcers. Diagn. Microbiol. Infect. Dis..

[17] Lipsky B.A., Holroyd K.J., Zasloff M. (2008). Topical versus Systemic Antimicrobial Therapy for Treating Mildly Infected Diabetic Foot Ulcers: A Randomized, Controlled, Double-Blinded, Multicenter Trial of Pexiganan Cream. Clin. Infect. Dis..

[18] Roeder B., van Gils C.C., Maling S. (2000). Antibiotic Beads in the Treatment of Diabetic Pedal Osteomyelitis. J. Foot. Ankle. Surg..

[19] Schade V.L., Roukis T.S. (2010). The Role of Polymethylmethacrylate Antibiotic-Loaded Cement in Addition to Debridement for the Treatment of Soft Tissue and Osseous Infections of the Foot and Ankle. J. Foot. Ankle. Surg..

[20] En A., Sanitarios P. (2016). Cementos Óseos Con Antibiótico. Panor. Actual del Medicam..

[21] Marson B.A., Grindlay D.J.C., Ollivere B.J., Deshmukh S.R., Scammell B.E. (2018). A Systematic Review of Local Antibiotic Devices Used to Improve Wound Healing Following the Surgical Management of Foot Infections in Diabetics. Bone. Jt. J..

[22] Sen P., Demirdal T., Emir B. (2019). Meta-Analysis of Risk Factors for Amputation in Diabetic Foot Infections. Diabetes Metab. Res. Rev..

[23] Varga M., Sixta B., Bem R., Matia I., Jirkovska A., Adamec M. (2014). Application of Gentamicin-Collagen Sponge Shortened Wound Healing Time after Minor Amputations in Diabetic Patients—a Prospective, Randomised Trial. Arch. Med. Sci..

[24] Uçkay I., Kressmann B., di Tommaso S., Portela M., Alwan H., Vuagnat H., Maître S., Paoli C., Lipsky B.A. (2018). A Randomized Controlled Trial of the Safety and Efficacy of a Topical Gentamicin–Collagen Sponge in Diabetic Patients with a Mild Foot Ulcer Infection. SAGE Open Med..

[25] Lipsky B.A., Kuss M., Edmonds M., Reyzelman A., Sigal F. (2012). Topical Application of a Gentamicin-Collagen Sponge Combined with Systemic Antibiotic Therapy for the Treatment of Diabetic Foot Infections of Moderate Severity. J. Am. Podiatr. Med. Assoc..

[26] Uçkay I., Kressmann B., Malacarne S., Toumanova A., Jaafar J., Lew D., Lipsky B.A. (2018). A Randomized, Controlled Study to Investigate the Efficacy and Safety of a Topical Gentamicin-Collagen Sponge in Combination with Systemic Antibiotic Therapy in Diabetic Patients with a Moderate or Severe Foot Ulcer Infection. BMC Infect. Dis..

[27] Hosch J., Quiroga C., Bosma J., Peters E.J.G., Armstrong D.G., Lavery L.A. (1997). Outcomes of Transmetatarsal Amputations in Patients with Diabetes Mellitus. J. Foot. Ankle. Surg..

[28] Durham J.R., McCoy D.M., Sawchuk A.P., Meyer J.P., Schwarcz T.H., Eldrup-Jorgensen J., Flanigan D.P., Schuler J.J. (1989). Open Transmetatarsal Amputation in the Treatment of Severe Foot Infections. Am. J. Surg..

[29] Armstrong D.G., Findlow A.H., Oyibo S.O., Boulton A.J.M. (2001). The Use of Absorbable Antibiotic-Impregnated Calcium Sulphate Pellets in the Management of Diabetic Foot Infections. Diabet. Med..

[30] Krause F.G., deVries G., Meakin C., Kalla T.P., Younger A.S. (2009). Outcome of Transmetatarsal Amputations in Diabetics Using Antibiotic Beads. Foot. Ankle. Int..

[31] Niazi N.S., Drampalos E., Morrissey N., Jahangir N., Wee A., Pillai A. (2019). Adjuvant Antibiotic Loaded Bio Composite in the Management of Diabetic Foot Osteomyelitis—A Multicentre Study. Foot.

[32] Chatzipapas C., Kougioumtzis I.E., Karaglani M., Panagopoulos P., Panopoulou M., Papazoglou D., Drosos G.I., Papanas N. (2022). Local Antibiotic Delivery Systems in the Surgical Treatment of Diabetic Foot Osteomyelitis: Again, No Benefit?. Int. J. Low Extrem Wounds.

[33] Dalla Paola L., Carone A., Morisi C., Cardillo S., Pattavina M. (2015). Conservative Surgical Treatment of Infected Ulceration of the First Metatarsophalangeal Joint With Osteomyelitis in Diabetic Patients. J. Foot Ankle Surg..

[34] Elmarsafi T., Oliver N.G., Steinberg J.S., Evans K.K., Attinger C.E., Kim P.J. (2017). Long-Term Outcomes of Permanent Cement Spacers in the Infected Foot. J. Foot Ankle Surg..

[35] Jogia R.M., Modha D.E., Nisal K., Berrington R., Kong M.-F. (2015). Use of Highly Purified Synthetic Calcium Sulfate Impregnated With Antibiotics for the Management of Diabetic Foot Ulcers Complicated by Osteomyelitis. Diabetes Care.

[36] Melamed E.A., Peled E. (2012). Antibiotic Impregnated Cement Spacer for Salvage of Diabetic Osteomyelitis. Foot Ankle Int..

[37] Qin C.-H., Zhou C.-H., Song H.-J., Cheng G.-Y., Zhang H.-A., Fang J., Tao R. (2019). Infected Bone Resection plus Adjuvant Antibiotic-Impregnated Calcium Sulfate versus Infected Bone Resection Alone in the Treatment of Diabetic Forefoot Osteomyelitis. BMC Musculoskelet Disord.

[38] Morley R., Lopez F., Webb F. (2016). Calcium Sulphate as a Drug Delivery System in a Deep Diabetic Foot Infection. Foot.

[39] Patil P., Singh R., Agarwal A., Wadhwa R., Bal A., Vaidya S. (2021). Diabetic Foot Ulcers and Osteomyelitis: Use of Biodegradable Calcium Sulfate Beads Impregnated With Antibiotics for Treatment of Multidrug-Resistant Organisms. Wounds.

[40] Roukis T.S., Schweinberger M.H., Schade V.L. (2010). V-Y Fasciocutaneous Advancement Flap Coverage of Soft Tissue Defects of the Foot in the Patient at High Risk. J. Foot Ankle Surg..

[41] Mendame Ehya R.E., Zhang H., Qi B., Yu A. (2021). Application and Clinical Effectiveness of Antibiotic-Loaded Bone Cement to Promote Soft Tissue Granulation in the Treatment of Neuropathic Diabetic Foot Ulcers Complicated by Osteomyelitis: A Randomized Controlled Trial. J. Diabetes Res..

[42] Sun Y.-W., Li L., Zhang Z.-H. (2022). Antibiotic-Loaded Bone Cement Combined with Vacuum-Assisted Closure Facilitating Wound Healing in Wagner 3–4 Diabetic Foot Ulcers. Int. J. Low Extrem Wounds.

[43] Aragón-Sánchez J., Lázaro-Martínez J.L., Alvaro-Afonso F.J., Molinés-Barroso R. (2015). Conservative Surgery of Diabetic Forefoot Osteomyelitis: How Can i Operate on This Patient without Amputation?. Int. J. Low. Extrem. Wounds.

[44] Winkler E., Schöni M., Krähenbühl N., Uçkay I., Waibel F.W.A. (2022). Foot Osteomyelitis Location and Rates of Primary or Secondary Major Amputations in Patients With Diabetes. Foot Ankle Int..

[45] Drampalos E., Mohammad H.R., Kosmidis C., Balal M., Wong J., Pillai A. (2018). Single Stage Treatment of Diabetic Calcaneal Osteomyelitis with an Absorbable Gentamicin-Loaded Calcium Sulphate/Hydroxyapatite Biocomposite: The Silo Technique. Foot.

[46] Maurer S.M., Hepp Z.S., McCallin S., Waibel F.W.A., Romero F.C., Zorman Y., Lipsky B.A., Uçkay İ. (2022). Short and Oral Antimicrobial Therapy for Diabetic Foot Infection: A Narrative Review of Current Knowledge. J. Bone Jt. Infect..

[47] Pham T.-T., Gariani K., Richard J.-C., Kressmann B., Jornayvaz F.R., Philippe J., Lipsky B.A., Uçkay I. (2022). Moderate to Severe Soft Tissue Diabetic Foot Infections. Ann. Surg..

[48] Gariani K., Pham T.-T., Kressmann B., Jornayvaz F.R., Gastaldi G., Stafylakis D., Philippe J., Lipsky B.A., Uçkay L. (2021). Three Weeks Versus Six Weeks of Antibiotic Therapy for Diabetic Foot Osteomyelitis: A Prospective, Randomized, Noninferiority Pilot Trial. Clin. Infect. Dis..

[49] Panagopoulos P., Drosos G., Maltezos E., Papanas N. (2015). Local Antibiotic Delivery Systems in Diabetic Foot Osteomyelitis. Int. J. Low Extrem Wounds.

[50] Dudareva M., Kümin M., Vach W., Kaier K., Ferguson J., McNally M., Scarborough M. (2019). Short or Long Antibiotic Regimes in Orthopaedics (SOLARIO): A Randomised Controlled Open-Label Non-Inferiority Trial of Duration of Systemic Antibiotics in Adults with Orthopaedic Infection Treated Operatively with Local Antibiotic Therapy. Trials.

[51] Page M.J., McKenzie J.E., Bossuyt P.M., Boutron I., Hoffmann T.C., Mulrow C.D., Shamseer L., Tetzlaff J.M., Akl E.A., Brennan S.E. (2021). The PRISMA 2020 Statement: An Updated Guideline for Reporting Systematic Reviews. BMJ.

[52] Baethge C., Goldbeck-Wood S., Mertens S. (2019). SANRA—A Scale for the Quality Assessment of Narrative Review Articles. Res. Integr. Peer Rev..

