# Supplementary material for: Local Antibiotics in the Treatment of Diabetic Foot Infections: A Narrative Review
Source: Antibiotics (Basel). 2023 Jan 9;12(1):124. doi: 10.3390/antibiotics12010124 (PMC9854429; doi:10.3390/antibiotics12010124)
Supplement: Supplementary file 1 [file antibiotics-12-00124-s001.zip › antibiotics-2131881-supplementary.pdf]

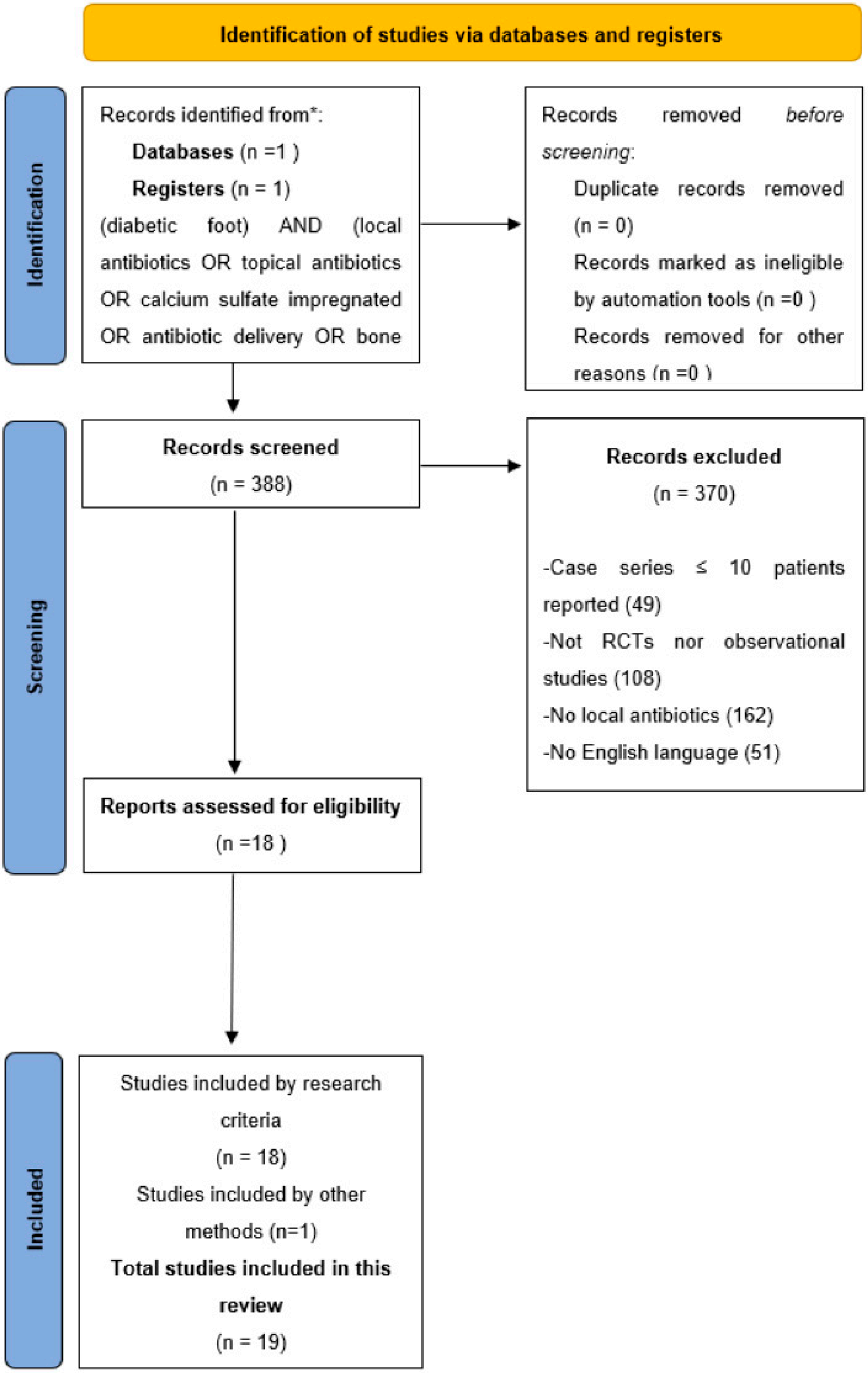

Figure S1. Flowchart of included studies.

File S1. Search strategy in PubMed

Concept 1: Diabetic patients with foot problems
1. 'diabetic foot' (15,598)
Concept 2: Topical and Local antibiotics
2. 'local antibiotics' (34,550)
3. 'topical antibiotics' (11,841)
4. 'calcium sulfate impregnated' (163)
5. 'antibiotic delivery' (21,765)
6. 'bone cement' (22,811)
7. #2 OR #3 OR #4 OR #5 OR #6 (84,641)
Concept 1 AND Concept 2
8. #1 AND #7 (388)
